# AI-Based Radiological Imaging for HCC: Current Status and Future of Ultrasound

**DOI:** 10.3390/diagnostics11020292

**Published:** 2021-02-12

**Authors:** Hitoshi Maruyama, Tadashi Yamaguchi, Hiroaki Nagamatsu, Shuichiro Shiina

**Affiliations:** 1 Department of Gastroenterology, Juntendo University, 2-1-1, Hongo, Bunkyo-ku, Tokyo 113-8421, Japan; h-nagamatsu@juntendo.ac.jp (H.N.); s.shiina@gmail.com (S.S.); 2 Center for Frontier Medical Engineering, Chiba University, 1-33 Yayoicho, Inage, Chiba 263-8522, Japan; yamaguchi@faculty.chiba-u.jp

**Keywords:** Hepatocellular carcinoma, ultrasound, radiomics, artificial intelligence

## Abstract

Hepatocellular carcinoma (HCC) is a common cancer worldwide. Recent international guidelines request an identification of the stage and patient background/condition for an appropriate decision for the management direction. Radiomics is a technology based on the quantitative extraction of image characteristics from radiological imaging modalities. Artificial intelligence (AI) algorithms are the principal axis of the radiomics procedure and may provide various results from large data sets beyond conventional techniques. This review article focused on the application of the radiomics-related diagnosis of HCC using radiological imaging (computed tomography, magnetic resonance imaging, and ultrasound (B-mode, contrast-enhanced ultrasound, and elastography)), and discussed the current role, limitation and future of ultrasound. Although the evidence has shown the positive effect of AI-based ultrasound in the prediction of tumor characteristics and malignant potential, posttreatment response and prognosis, there are still a number of issues in the practical management of patients with HCC. It is highly expected that the wide range of applications of AI for ultrasound will support the further improvement of the diagnostic ability of HCC and provide a great benefit to the patients.

## 1. Introduction

Liver cancer is the sixth most common cancer by incidence and the fourth most common cause of cancer-related mortality worldwide [1]. Hepatocellular carcinoma (HCC) represents primary liver cancer and develops mainly in patients with chronic liver diseases. Although we are in an era of the possible control of viral activities, which is the major factor for hepatocarcinogenesis, HCC is still seriously problematic [2]. The development of HCC limits the prognosis as well as the quality of life of patients. Its management should be properly conducted based on an accurate diagnosis.

As shown in recent international guidelines, the identification of the stage and patient background/condition need to be properly assessed to select the appropriate treatment [3,4,5]. In general, liver function reserve by Child–Pugh score and degree of tumor progression (tumor number and size, vascular invasion, extrahepatic metastasis), evaluated by imaging modalities such as contrast-enhanced computed tomography (CT) and/or magnetic resonance imaging (MRI), are key factors to decide the appropriate treatment from multiple options, such as surgical resection, local ablation (radiofrequency ablation or microwave ablation under ultrasound (US)/CT guidance), percutaneous ethanol injection, transcatheter arterial chemoembolization (TACE), image-guided high-dose-rate brachytherapy, chemotherapy using molecular targeted agents, liver transplantation and best supportive care.

Because of the advantages of noninvasiveness and possible real-time observation, ultrasound (US) is the most frequently used imaging modality for liver diseases. This simple technique is applied for detailed examination as well as for a first-line approach. After the clinical use of the first-generation microbubble-based contrast agent Levovist, several second-generation contrast agents have become available in the abdominal field [6]. A specific harmonic mode enables high sensitivity for microbubble detection while being less affected by artefacts compared with the Doppler mode. Based on this background, contrast-enhanced US (CEUS) has become popular due to its capacity for stable and real-time observation with the improved detectability of peripheral blood flow under vascular-phase imaging. In addition, the in vivo properties of contrast agents vary, and microbubbles of Sonazoid accumulate in reticuloendothelial tissue, such as Kupffer cells. Postvascular phase images using this property are effective for detecting occult hepatic lesions, differentiating between benign and malignant lesions, and evaluating therapeutic results. However, there are still several limitations in the diagnostic performance, such as with deeply located lesions and blind spots [6,7].

Radiomics is a technology based on the quantitative extraction of image characteristics from radiological imaging modalities [8]. The data, so-called “radiomics features”, are used to predict clinical endpoints such as histological findings, malignant potential, therapeutic response, and prognosis, by using artificial intelligence (AI) algorithms, which may be beyond conventional techniques [9]. In fact, investigators have shown the effect of radiomics signatures in detecting the risk of lymph node metastasis in patients with colorectal and bladder cancer [10,11]. Additionally, this may be a predictive indicator for progression-free and overall survival in patients with malignant diseases [12,13].

Changes of morphological and hemodynamic features, depending on the cellular differentiation and variety of malignant grade, are the unique aspect and characteristic features of HCC [2,3,4,5]. These are the most important clinical issues of the diagnosis of HCC, which have not been solved by current imaging methods, and therefore, the solution by radiomics is highly expected.

Against these backgrounds, this review article focuses on the application of the radiomics-based diagnosis of HCC using US. We also reviewed recent studies employing radiomics based on CT and MRI studies for HCC, and discussed the advantages/disadvantages of US in this manner, as well as future directions.

## 2. Deep Learning

The definition of deep learning is “a particular kind of machine learning that achieves great power and flexibility by learning to represent the world as a nested hierarchy of concepts, with each concept defined in relation to simpler concepts, and more abstract representations computed in terms of less abstract ones.” [14]. Deep-learning methods indicate the representation created by composing simple but nonlinear modules that each transform the representation at one level (starting with the raw input) into a representation at a higher level [15].

Recent studies suggest that transfer learning on a deep learning model trained natively on US images, and fine-tuning the model on a new data set obtained from a different medical center and/or a different device to overcome the limitations of the US data set, such as those obtained from a single medical center and a single US device, is problematic [16]. Additionally, real-time feedback to the sonographer during image acquisition by machine learning systems is recommended to address poor reproducibility [17]. Moreover, the automatic control of US examination, settings for image quality control, and region of interest (ROI) selection may be useful against a wide range of intra- and interobserver/operator variability in image acquisition.

Regardless, deep learning should continue to make progress and may solve problems that have resisted the best attempts of the artificial intelligence community for many years.

## 3. Workflow

**Figure 1:**
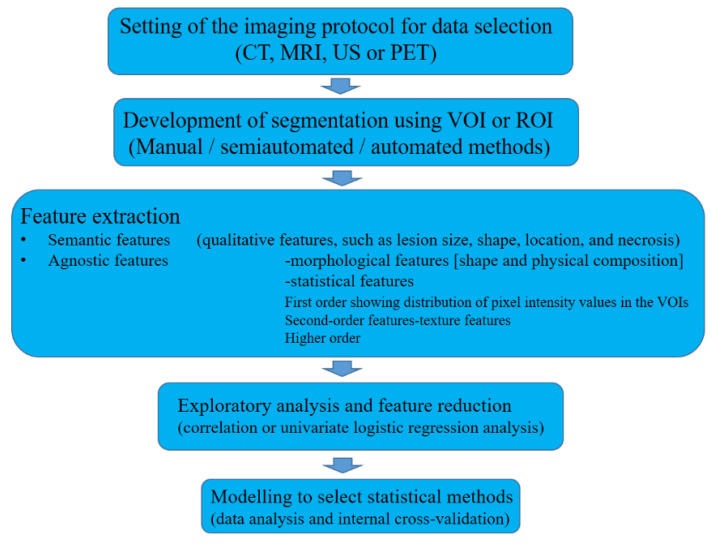
Diagram of workflow. CT, computed tomography; MRI, magnetic resonance imaging; US, ultrasound; PET, positron emission tomography modality; VOI, volume of interest; ROI, region of interest.

Radiomics is a multistep process that requires optimization, standardization, quality control, and algorithm refinement (Figure 1). The initial step is the setting of the imaging protocol for data selection. Any CT, MRI, US or positron emission tomography modality could be used; however, the first two are frequently applied because of their reproducibility and comparability. Next is the development of segmentation using volume of interest (VOI) or ROI with manual, semiautomated or automated methods. The third process is feature extraction, which consists of semantic features (qualitative features, such as lesion size, shape, location, and necrosis) and agnostic features (defined by an advanced mathematical algorithm). The former depends on the observer’s skill and experience, and the latter includes two kinds of features: morphological features (shape and physical composition) and statistical features (first-order, showing the distribution of pixel intensity values in the VOIs, second-order, showing texture features, and higher order). The fourth step is exploratory analysis and feature reduction with the use of correlation or univariate logistic regression analysis, and the final step is modeling to select statistical methods for data analysis and internal cross-validation [9]. A recent study has shown the possibility of using a deep learning algorithm to segment the liver and HCCs automatically, suggesting a more workflow-efficient and clinically realistic implementation of the Liver Imaging Reporting and Data System [18].

## 4. Diagnosis of HCC by CT/MRI Based Radiomics

### 4.1. Characterization

Multistep carcinogenesis is the representative progression of HCC, from dysplastic nodules to moderately differentiated HCC [19]. As high-grade dysplastic nodules show strong malignant potential, they are recognized as precursors of HCC. Meanwhile, in cirrhosis patients, regenerative nodules (RNs) are also frequently detected as typical benign nodules as a result of remodeling in the liver. In addition, there is a possibility of the occurrence of other kinds of hepatic lesions: benign lesions such as hemangioma, focal nodular hyperplasia (FNH) and angiomyolipoma, and malignant lesions such as cholangiocarcinoma and metastasis. Thus, the noninvasive characterization of hepatic lesions is a clinical challenge.

For the differentiation between HCC and benign hepatic lesions, a CT-based radiomics nomogram, which incorporated the rad-score and clinical factors (age, hepatitis B virus infection, and enhancement pattern), showed an area under the receiver operating characteristic curve (AUC) of 0.917 for differentiating FNH from HCC in a study performed with 156 patients (FNH 55, HCC 101) [20]. An MRI-based study in 369 patients with 446 lesions (HCC 222, hemangioma 224) reported an AUC of 0.89 (sensitivity 0.822, specificity 0.714) for differentiating between HCC and hemangioma using images with in-phase, out-phase, T2-weighted, and diffusion-weighted imaging sequences. This AUC was significantly higher than that of the less experienced radiologist (2 years of experience) (sensitivity 0.625, specificity 0.779; AUC 0.702, *p* < 0.05); however, it showed no significant difference from the experienced radiologist (10 years of experience) (sensitivity 0.915, specificity 0.901; AUC 0.908, *p* > 0.05) [21]. In addition, according to a more recent study with both CT and MRI, fusion models that simultaneously integrated clinical characteristics achieved average AUCs of 0.966 (CT) and 0.971 (MRI), with 10-fold cross-validation to differentiate hepatic epithelioid angiomyolipoma from HCC and FNH [22]. Furthermore, a multicenter retrospective cohort study performed in 178 cirrhosis patients (with indeterminate liver nodules including other malignant lesions (cholangiocarcinoma <CC> and metastasis), regenerative nodule, hemangioma and FNH) reported an AUC of 0.66 to diagnose HCC using triphasic contrast-enhanced CT, and suggested the benefit of AI to enhance clinicians’ decisions by identifying a subgroup of patients with high HCC risk [23].

Another study also stressed the effect of using radiomics: contrast-enhanced MRI and precontrast and portal-phase CT exhibited good performance in the differentiation of HCC from non-HCC (AUC of 0.79 to 0.81 for MRI and AUC of 0.81 and 0.71 for CT). The rates of the misdiagnosis of combined hepatocellular CC as HCC or CC using radiologists’ readings were 69% by CT and 58% by MRI [24]. These data may ground strong recommendations for the application of radiomics analysis, with future validation, for the preoperative diagnosis of liver cancer and for optimal treatment decisions regarding liver resection and transplantation.

### 4.2. Malignant Potential

The degree of malignant potential is an important issue for the therapeutic direction and posttreatment outcomes of HCC patients. Two radiomics-based studies examined the histological grade of HCC. The first study, performed in 170 patients with HCC (training group 125, test group 45) using T1-weighted imaging and T2-weighted imaging with clinical factors (age, sex, tumor size, alpha fetoprotein (AFP) level, history of hepatitis B, hepatocirrhosis, portal vein tumor thrombosis, portal hypertension and pseudocapsule), reported an AUC of 0.800 for the prediction of the histological grading of HCC presented by Edmondson grades [25]. A more recent study using contrast-enhanced CT performed in 297 HCC patients demonstrated an AUC of 0.8014 (sensitivity, 0.65; specificity, 0.73; accuracy, 0.7) to differentiate between low- and high-grade HCC by radiomics signatures in association with clinical factors [26].

Investigators have also examined the prediction of protein markers by using radiomics. Cytokeratins are intermediate filament proteins that are expressed in epithelial cells; cytokeratin 8 and cytokeratin 18 are present in hepatocytes, and cytokeratin 7 and cytokeratin 19 are present in cholangiocytes. HCC tumors express the latter (cytokeratin 7 and cytokeratin 19) as biliary-specific markers, and cytokeratin 19-positive HCC is considered to be related to clinical aggressiveness, such as tumor invasion, lymph node metastasis, and poor prognosis after resection and liver transplantation. The study performed by Wang et al. used gadoxetic acid-enhanced MR images with a clinical model (AFP, irregular tumor margin, and arterial rim enhancement) in 227 patients with single HCC (training set 159 patients, a time-independent validated set 68 patients), and found a C-index of 0.846, with a sensitivity of 0.769 and a specificity of 0.818, for the identification of the cytokeratin 19 status of HCC [27].

Glypican 3 is a cell-surface protein with roles in cellular growth, migration, and differentiation [28,29]. It is not present in benign liver tissue but is highly expressed in HCC tissues, and it shows a close relationship with metastasis/recurrence in patients with HCC after surgery [30]; therefore, it represents a marker for poor prognosis. For a study using a combined nomogram integrating independent clinical risk factors, AFP and radiomics signatures with contrast-enhanced MRI, the AUCs were 0.926 (training cohorts) and 0.914 (validation cohorts) for identifying HCC with glypican 3 positivity [31]. Geng et al. examined the efficacy of radiomics using MRI-based susceptibility weighted imaging, and found AUCs of 0.905, 0.837, 0.800 and 0.760 for diagnosing patients with positive cytokeratin 19, positive cytokeratin 7, high histopathologic grade and positive glypican 3 [32].

The expression of the human Ki-67 protein is closely related to cell proliferation. The antigen is demonstrated in the nucleus during interphase; however, most of the protein is relocated to the surface of the chromosomes during mitosis. That is, the Ki-67 protein is present during all active phases of the cell cycle (G(1), S, G(2), and mitosis), but is absent from resting cells (G(0)) [33]. Thus, the Ki-67 protein has been widely used as a proliferation marker for human tumor cells [34]. For the assessment of potential malignancy, a higher Ki-67 level is considered a marker for fast progression and poor prognosis in malignant diseases, such as HCC, breast cancer and bladder cancer [35,36,37]. A recent study using radiomics data with contrast-enhanced CT showed AUCs of 0.777–0.836 to predict Ki-67 status in HCC patients, suggesting the possibility of radiomics analysis as a noninvasive marker of the cellular proliferation of HCC [38]. Taken together, MRI- or CT-related radiomics models may be available to predict the degree and malignant severity of HCC.

### 4.3. Microvascular Invasion

Microvascular invasion (MVI) is defined by tumor invasion into the intravascular space [39]. The incidence of MVI in resected liver specimens is reported to range from 15.0% to 57.1% [40]. Studies have shown that the presence of MVI strongly suggests a more severe malignant degree of HCC because of the sign of early recurrence [39] and worse prognosis after surgical resection [41] or transplantation [42]. However, the presence of MVI is diagnosed by histological examination using resected specimens after surgical procedures; therefore, undetermined preoperative judgement of the therapeutic direction has been a clinical problem.

Against this background, MVI is a well-documented target of AI-based studies (Table 1). Four studies reported the effect of combined models incorporating the contrast-enhanced CT radiomics signature and the effective clinical factors to predict MVI status; an AUROC of 0.801 (C-indices 0.820) was reported in a study performed in 157 patients with histologically proven HCC and with a clinical model including age, maximum tumor diameter, AFP and hepatitis B virus antigen [43], and a C-index of 0.844 was reported in a study performed in 304 patients with HCC and with clinical factors including AFP, hypoattenuating halos, arterial peritumoral enhancement, and nonsmooth tumor margin [44]. Zhang et al. showed the ability of CT-based radiomics to predict both MVI status (positive vs. negative) and risk (high vs. low), with an AUC of 0.796 for MVI status and an AUC of 0.740 for MVI risk. Furthermore, the MVI status classifier significantly stratified patients for short overall survival or early tumor recurrence [45]. A more recent study focused on predictive models using eXtreme Gradient Boosting (XGBoost) and deep learning based on CT images. The AUROCs of the radiomics–radiological–clinical (RRC) model and three-dimensional convolutional neural network model were 0.887 and 0.906, respectively (*p* = 0.83). Interestingly, moreover, based on the MVI status predicted by the RRC and three-dimensional convolutional neural network models, the mean recurrence-free survival was significantly better in the predicted MVI-negative group than in the predicted MVI-positive group (RRC Model: 69.95 vs. 24.80 months, *p* < 0.001; three-dimensional convolutional neural network model: 64.06 vs. 31.05 months, *p* = 0.027) [46].

Meanwhile, three studies demonstrated the effect of a combined model incorporating the contrast-enhanced MRI radiomics signature and clinical factors. The first study performed in 267 HCC patients reported an AUC of 0.858, with a sensitivity of 80.77% and a specificity of 68.09%, using precontrast and contrast-enhanced MRI with gadopentetate dimeglumine and two clinical factors (arterial peritumoral enhancement and AFP) [51]. According to the second study performed in patients with pathologically confirmed HCC (training cohort 146 patients, validation cohort 62 patients), the AUC was 0.861 (C-index of 0.864), with a sensitivity of 89.5%, a specificity of 81.4%, and an accuracy of 83.9%, using precontrast and contrast-enhanced MRI with gadoxetic acid and clinical factors including AFP, nonsmooth tumor margin, and arterial peritumoral enhancement [50]. Third, Nebbia et al. showed the highest AUC (0.867) and accuracy (79.68%) in predicting MVI status by T2 and portal venous sequences of gadopentetic acid-enhanced MRI [52]. Based on these data, the preoperative identification of MVI by the application of an AI-based combined model using CT/MRI may be promising for deciding the appropriate therapeutic direction, leading to a reduction in recurrence and an improvement in posttreatment outcomes and prognosis.

## 5. Prediction of Therapeutic Response by CT/MRI-Based Radiomics

### 5.1. Transcatheter Arterial Chemoembolization (TACE)

Two retrospective studies using MRI reported the effect of AI-based analysis for the prediction of the response to TACE. The first study performed in 36 HCC patients in the US showed preprocedural prediction with an overall accuracy of 78% (sensitivity 62.5%, specificity 82.1%, positive predictive value 50.0%, negative predictive value 88.5%), using combined clinical patient data with baseline contrast-enhanced multiphasic MRI and with gadopentetate dimeglumine [53]. Another study with 84 HCC patients from China revealed that the model based on diffusion-weighted image features showed an AUC (b = 0, 0.786; b = 500, 0.729), followed by T2-weighted image features (0.729) and apparent diffusion coefficient images (0.714). Additionally, the radiomics signature was an independent parameter of progressive disease (PD), while clinical information had no significance in the PD group [54].

### 5.2. Immunotherapy

The intratumor immunoscore, which assesses the density of CD3+ and CD8+ T cells, is effective in determining the application of immunotherapy in HCC patients [55,56]. A retrospective study in 207 HCC patients who underwent hepatectomy demonstrated that the combined radiomics (gadolinium ethoxybenzyl-diethylenetriaminepentaacetic acid-enhanced MRI) and clinical model (AFP, gamma-glutamyltransferase, aspartate aminotransferase) showed the highest ability to predict the immunoscore, with an AUC of 0.934, a sensitivity of 0.846, a specificity of 0.841, an accuracy of 0.842, a positive predictive value of 0.611 and a negative predictive value of 0.949 [57]. According to a more recent retrospective study performed in 48 patients with HCC who underwent hepatic resection or transplant, MRI-based radiomics features correlated with immunohistochemical cell type markers for T-cells (CD3), macrophages (CD68), endothelial cells (CD31), and the expression of immunotherapy targets programmed death-ligand 1, at the protein level (r = 0.41–0.47, *p* < 0.029), and programmed death receptor 1 and cytotoxic T-lymphocyte associated protein 4 at the mRNA expression level (r = −0.48–0.47, *p* < 0.037). In addition, the model showed diagnostic performance (AUC 0.76–0.80, *p* < 0.043) for the assessment of early HCC recurrence, although immune profiling and genomic features did not (*p* = 0.098–0929) [58].

These data suggest the effect of radiomics-based data to help make treatment decisions by the noninvasive prediction of the response to TACE and immune-oncologic features. Thus, if the therapeutic direction is considered to be unresponsive to TACE, it may be changed to the use of molecular targeted agents (i.e., sorafenib). However, the practical benefit of radiomics-based findings needs to be validated by additional studies in a prospective setting.

## 6. Prediction of Posttreatment Recurrence and Prognosis by CT/MRI-Based Radiomics

The prediction of posttreatment outcomes is a pivotal issue in the management of patients with HCC. Investigators have performed radiomics-based studies regarding the prediction of early recurrence, and most of them have shown better results in combined models with radiomics and clinical data than in radiomics data alone [59,60,61,62,63,64,65,66,67,68]. The diagnostic abilities to predict early recurrence after surgical or nonsurgical treatment ranged from 0.785 to 0.89, according to the AUC, and 0.743 to 0.809 according to the C-index, with the use of CT-based radiomics data (Table 2).

Prognosis is another important posttreatment outcome that requires proper prediction. Four retrospective studies reported the effect of the radiomics-based model on predicting survival after TACE. A first study performed in 66 HCC patients examined the effects of three models using pretreatment contrast-enhanced CT, and found that the combined model using clinical data (Child–Pugh score, AFP, and HCC size) was a better predictor of survival (hazard ratio, 19.88; *p* < 0.0001) than the others for predicting overall survival [70]. Similar data for predicting survival after TACE were presented by Meng et al.; a combined radiomics–clinic model using contrast-enhanced CT showed C-indices of 0.73 and 0.70 in the training and testing cohorts, respectively [71]. The integration of multimodal data with contrast-enhanced CT also appears effective in predicting prognosis after TACE, showing an accuracy (concordance index) of 0.73 and AUCs ranging from 0.85 to 0.90 in predicting 3- to 10-year survival [72]. The last study in 184 HCC patients used pretreatment contrast-enhanced MRI with gadodiamide for recurrence-free survival, and the combined model was the best with a C-index of 0.802 [73].

For the prediction of post-hepatectomy prognosis, three retrospective studies reported the usefulness of radiomics-based combined data [74,75,76], and Zhang et al. supported those findings with a prospective study in which there was an improved predictive performance, with a C-index of 0.92 compared to the clinic–radiological model (C-index, 0.86, *p* = 0.039) or the combined rad-score (C-index, 0.88, *p* = 0.016) in their prospective study [77].

Post-hepatectomy liver failure is a serious consequence in clinical practice. According to a retrospective study of 112 HCC patients, the contrast-enhanced CT-based radiomics score could predict posthepatectomy liver failure with an AUC of 0.762. Additionally, the individual predictive nomogram that included the radiomics-based score, model for end-stage liver disease (MELD) and performance status showed that the AUC of 0.896 of the nomogram discrimination was superior to those of the others [78].

Thus, comprehensive analysis using AI techniques seems useful to predict posttreatment outcomes and prognoses. However, most of the studies were performed in retrospective settings, and TACE and surgical resection were the majority of the treatments of choice. Further validation is warranted in a large cohort prospective manner, including various treatment options, such as local ablation and molecular targeted therapy.

## 7. Radiomics-Based US for the Diagnosis of HCC

Preoperative diagnosis of primary liver cancer, particularly differentiation between HCC, combined hepatocellular–cholangiocarcinoma (cHCC-CC), and intrahepatic cholangiocarcinoma (CC), is pivotal in decisions regarding patient management. A recent retrospective study analyzed US images of 668 patients with primary liver cancer, consisting of 531 HCC patients, 48 cHCC-CC patients, and 89 CC patients, and found that the overall performance of the radiomics model in identifying different histopathological types of primary liver cancer yielded AUCs of 0.854 (training cohort) and 0.775 (test cohort) in the HCC vs. non-HCC radiomics model, and 0.920 (training cohort) and 0.728 (test cohort) in the CC vs. cHCC-CC radiomics model [79]. Furthermore, a multicenter study performed in 13 hospitals, including 2143 patients (24343 sonograms), has shown that deep convolutional neural network of US may have the potential to assist less experienced radiologists in improving their performance and lowering their dependence on sectional imaging in liver cancer diagnosis [47].

The prediction of malignant potential (posttreatment recurrence and poor prognosis) is a key issue in the practical daily care of patients with HCC, and some protein markers and the presence/absence of MVI are used for this purpose. For the former, although the sample size was small (47 patients with HCC), a radiomics-based study including B-mode, share wave elastography (SWE) and viscosity imaging demonstrated high diagnostic ability (AUC 0.94) to predict Ki-67, which is a marker to indicate the poor prognosis of several malignant diseases [48]. For the latter, two retrospective studies using B-mode US findings showed AUCs of 0.731 and 0.726 (0.806 for differentiation between M1 and M2) in predicting MVI [49,80]. In addition, Yao et al. reported a higher diagnostic ability (AUC 0.98) achieved by radiomics-based multimodal US images [48].

A retrospective study performed in 130 HCC patients using US (B-mode, CEUS with SonoVue) for the prediction of the response to TACE demonstrated that the AUCs of radiomics data were 0.93 by CEUS, 0.80 by time–intensity curve, and 0.81 by B-mode, showing significant differences between CEUS and the other two (time–intensity curve, *p* = 0.034; B-mode, *p* = 0.039) [81]. For therapeutic strategy decisions, radiomics-based multimodal US images may also be useful for the prediction of the effect of programmed cell death-1, with an AUC of 0.97 [48].

A more recent study, performed in 419 patients examined by CEUS within 1 week before receiving radiofrequency ablation or surgical resection (radiofrequency ablation: 214, surgical resection: 205), analyzed the CEUS findings and found that 17.3% of radiofrequency ablation patients and 27.3% of surgical resection patients should swap their treatment; as a result, their average probability of 2-year progression-free survival would increase by 12% and 15%, respectively [82]. Moreover, dynamic CEUS radiomics performed well (AUC 0.84) in predicting the post-ablation early recurrence of HCC [69].

These data indicate the potential of radiomics-based US findings in facilitating optimized treatment selection for patients with HCC, by characterizing and predicting malignant potential and posttreatment outcomes (Table 3). However, as the numbers involved and the quality of the study were not sufficient, further evaluation is warranted.

## 8. Radiomics-Based US for the Diagnosis of Nontumor Liver Disease

There have been some attempts made to use US technology with deep learning regarding hepatic fibrosis and steatosis; as these are factors associated with hepatocarcinogenesis, the assessment of their degree is an important process for the evaluation of the risk of HCC development [83]. A study using the Inception-ResNet-v2 deep convolutional neural network showed an AUROC of 0.977 in detecting histologically proven fatty liver, defined by 5% or more hepatocytes with steatosis. The AUROC here was much higher than those of other modalities: 0.959 by the hepatorenal index method and 0.893 by the grey-level cooccurrence matrix algorithm. This suggests the novelty and utility of the automatic diagnosis of the amount of fat in the liver [84].

To detect advanced liver fibrosis, two methods, hepatic surface imaging and liver elasticity, have been investigated. For the former, a study employing a deep convolutional neural network model to extract features from US images resulted in the highest AUROC of 0.968, compared to other models, although the sample size was relatively small (44 control and 47 cirrhosis patients), and the diagnosis of cirrhosis was made clinically without histological assessment [85]. In the latter, a machine learning algorithm SWE color mapping image (Aixplorer ultrasonic system; SuperSonic Imagine, Aix-en-Provence, France) found a diagnostic ability of 87.3% accuracy, 93.5% sensitivity and 81.2% specificity, with an AUROC of 0.87 to differentiate between controls and subjects (chronic liver disease, defined by histologic examination), with 0.94 to 0.95 intraclass correlation coefficient indices of intraobserver variability [86]. Another multicenter study including a large patient population (398 patients with 1990 images) reported that deep learning radiomics of elastography showed the best overall performance in predicting liver fibrosis stages, compared with two dimensional SWE (2D-SWE) and biomarkers in patients with hepatitis B virus infection; the AUCs of deep learning radiomics of shear wave elastography were 0.97 for F4, 0.98 for ≥F3 and 0.85 for ≥F2, which were significantly better than other methods, except 2D-SWE in ≥F2 [87].

## 9. Radiomics in the Field of Point of Care US (POCUS)

In general, point-of-care US (POCUS) is defined as “US performed by the clinician providing care, which is brought to the location where the patient is receiving care ‘at the patient’s bedside,’ regardless of where that may be located (and even if the bed is just theoretical)” [88]. From the aspect of emergency medicine, the term indicates clinician- or physician-performed US at bedside, with focused and mobile settings [89]. US directly performed by the clinician who performs treatment provides prompt assessment of the patient condition, and may enhance the decision of management direction [90]. In the medical care of patients with HCC, biopsy and local treatment (ablation and percutaneous ethanol injection) are usually conducted under US guidance, which is operated by the hepatologist or radiologist. Furthermore, the operator must examine the target tumor location and adjacent structures such as vessels, bile ducts, gut, heart and diaphragm, and identify the presence of ascites, pleural effusion, portal vein thrombosis, and portal vein tumor thrombosis, for the recognition of the pretreatment patient condition and postprocedure complications.

An AI-based unique attempt has been made for POCUS using deep learning models with sets of thousands of images derived from a large database of US images, which include both normal and pathologic findings for the targeted conditions. With this background, deep learning models have been introduced as novel technologies to improve the accuracy and efficacy of POCUS imaging, by automated image interpretation and by matching various algorithms for specific patient conditions [91]. The results of these trials suggest the potential utility of deep learning radiomics of elastography as an alternative to the current SWE system in the noninvasive assessment of hepatic fibrosis.

## 10. Summary and Future Perspectives of AI-Based US

Obviously, US imaging may be the most commonly used imaging modality in the abdominal field. Investigators have shown the effect of the US-based radiomics approach in the prediction of tumor characteristics and malignant potential, by the assessment of the presence/absence of MVI and Ki-67, posttreatment response and prognosis. However, evidence has shown that the actual significance of AI-based US examination is still far behind the effect of CT or MRI. This may be discussed by the comparison of advantages and disadvantage between US and CT/MRI; the advantages of US are the simplicity, with near-noninvasiveness and possible real-time observation, and the lack of these points may be listed as the disadvantage of CT/MRI. Meanwhile, the disadvantages of US are operator- and patient-dependent variations. These factors may account for the poor objectivity and reproducibility of the US images; however, the higher objectivity and reproducibility of the data are the advantages of CT/MRI. That is, there is a trade-off relation between US and CT/MRI, and particularly, the disadvantages of US have a great influence on each step of the workflow of radiomics, which may also be linked to the small number of US-based radiomics studies.

The major future direction of US-based radiomics may depend on how to utilize US data, such as law data, cine clip data including multiple frames, and 3D data. Moreover, comprehensive integration using a broad spectrum of laboratory data may help to improve the potential of AI-related US examination. The advantage of radiomics is operator independence, which may resolve the shortcomings of US.

## 11. Conclusions

The evidence from studies employing radiomics analysis for HCC enhances the positive effect of using AI-based US analysis. It is highly expected that the wide range of applications of AI for US will support the further improvement of the diagnostic ability of HCC, and provide a great benefit to the patients.

## Figures and Tables

**Table 1:** Diagnostic ability of radiomics-based radiological imaging for microvascular invasion.

N	Image	AUC			Reference
		Clinical	Radiomics	Combined Model	
47	B-mode/SWE/VI	-	0.98	-	[47]
482	B-mode	0.634	0.731	-	[48]
	
322	B-mode	-	0.726 (presence/absence)	-	[49]
			0.806 (M1/M2)		
157	Contrast-enhanced CT	0.761	0.793	0.801	[43]
				(C-index 0.820)	
304	Contrast-enhanced CT	-	-	(C-index 0.844)	[44]
	
637	Contrast-enhanced CT	0.739 *	0.743 *	0.796 *	[45]
		0.529 **	0.7 **	0.74 **	
405	Contrast-enhanced CT	0.875	0.888	0.897	[46]
				(3D-CNN model 0.906)	
208	MRI	-	0.837	0.861	[50]
	
267	MRI	0.729	0.820	0.858	[51]
	
99	MRI	-	0.867	-	[52]
	

AUC, area under the receiver operating characteristic curve; SWE, share wave elastography; VI, viscosity imaging; CT, computed tomography; MRI, magnetic resonance imaging; 3D-CNN, three-dimensional convolutional neural network. * AUC for status of microvascular invasion; ** AUC for risk of microvascular invasion.

**Table 2:** Diagnostic ability of radiomics-based radiological imaging for posttreatment recurrence.

N	Image	Treatment	AUC			Reference
			Clinical	Radiomics	Combined Model	
215	Contrast-enhanced CT	RFA/PEI/TACE	0.781 *	0.817 *	0.836 *	[59]
203	Contrast-enhanced CT	Resection	-	0.79 *	-	[60]
		Ablation				
184	Contrast-enhanced CT	Ablation	0.649 **	0.791 **	0.809 **	[61]
155	MRI	Resection	0.814 *	0.728 *	0.841 *	[62]
129	MRI	Resection	-	-	-	[63]
470	Contrast-enhanced CT	Resection	0.739 **	0.801 **	-	[64]
133	Contrast-enhanced CT	Liver transplantation	0.675 **	0.743 **	0.785 **	[65]
295	Contrast-enhanced CT	Resection	0.71 ***	0.88 ***	-	[66]
114	Contrast-enhanced CT	Resection	0.63 *	0.89 *	0.89 *	[67]
		Ablation				
262	Contrast-enhanced CT	Resection	0.654 *	0.785 *	-	[68]
318	Contrast-enhanced US	Ablation	0.60	0.83	0.84	[69]

Diagnostic performance: * AUROC; ** C-index; *** Time-dependent AUC. CT, computed tomography; MRI, magnetic resonance imaging; RFA, radiofrequency ablation; PEI, percutaneous ethanol injection; TACE, transarterial chemoembolization.

**Table 3:** AI-based US studies for HCC.

N	Image	Target	AUC	Reference
668	B-mode	Histopathological types of primary liver cancer	HCC v. non-HCC 0.775 Intrahepatic cholangiocarcinoma vs. combined hepatocellular–cholangiocarcinoma 0.728	[79]
2143	B-mode	Liver cancer diagnosis	0.924 for focal hepatic lesions Sensitivity (86.5% vs. 76.1%, *p* = 0.0084) Specificity (85.5% vs. 76.9%, *p* = 0.0051) Both superior to 15-year skilled radiologists	[47]
47	B-mode, share wave elastography and viscosity imaging	Ki-67	0.94	[48]
47	B-mode, share wave elastography and viscosity imaging	Microvascular invasion	0.98	[48]
482	CEUS	Microvascular invasion	0.731	[49]
322	B-mode	Microvascular invasion	0.726 (0.806 for differentiation between M1 and M2) -	[80]
130	B-mode, CEUS	Response to TACE	0.93 by CEUS 0.80 by time–intensity curve (*p* = 0.034 vs. CEUS) 0.81 by B-mode (*p* = 0.039 vs. CEUS)	[81]
47	B-mode, share wave elastography and viscosity imaging	Programmed cell death-1	0.97	[48]
318	CEUS	Post-ablation early recurrence	0.84	[69]

CEUS, contrast-enhanced ultrasound.
